# Modified Dendritic cell-based T-cell expansion protocol and single-cell multi-omics allow for the selection of the most expanded and *in vitro*-effective clonotype via profiling of thousands of MAGE-A3-specific T-cells

**DOI:** 10.3389/fimmu.2024.1470130

**Published:** 2024-10-10

**Authors:** Sergey Sennikov, Marina Volynets, Saleh Alrhmoun, Roman Perik-Zavodskii, Olga Perik-Zavodskaia, Marina Fisher, Julia Lopatnikova, Julia Shevchenko, Kirill Nazarov, Julia Philippova, Alaa Alsalloum, Vasily Kurilin, Alexander Silkov

**Affiliations:** Laboratory of Molecular Immunology, Research Institute of Fundamental and Clinical Immunology, Novosibirsk, Russia

**Keywords:** MAGE-A3, ScRNA-seq, scTCR-seq, TCR, T-cell receptor repertoire, TCR T-cells, adoptive cell therapy, naturally-occurring T-cells

## Abstract

**Introduction:**

Adoptive cell therapy using TCR-engineered T-cells is one of the most effective strategies against tumor cells. The TCR T-cell approach has been well tested against a variety of blood neoplasms but is yet to be deeply tested against solid tumors. Among solid tumors, cancer-testis antigens are the most prominent targets for tumor-specific therapy, as they are usually found on cells that lie behind blood-tissue barriers.

**Methods:**

We have employed a novel efficient protocol for MAGE-A3-specific T-cell clonal expansion, performed single-cell multi-omic analysis of the expanded T-cells via BD Rhapsody, engineered a selected T-cell receptor into a lentiviral construct, and tested it in an *in vitro* LDH-cytotoxicity test.

**Results and discussion:**

We have observed a 191-fold increase in the MAGE-A3-specific T-cell abundance, obtained a dominant T-cell receptor via single-cell multi-omic BD Rhapsody data analysis in the TCRscape bioinformatics tool, and observed potent cytotoxicity of the dominant-clonotype transduced TCR T-cells against a MAGE-A3-positive tumor. We have demonstrated the efficiency of our T-cell enrichment protocol in obtaining potent anti-tumor T-cells and their T-cell receptors, especially when paired with the modern single-cell analysis methods.

## 1 Introduction

Melanoma-associated gene (MAGE) protein family were the first proteins identified from the class of cancer-testis antigens whose expression pattern was restricted to germline cells and immune-privileged testes and placenta cells (1). In tumor cells, expression of cancer-testis antigens is associated with apoptosis avoidance, increased viability, migration with subsequent metastasis, and angiogenesis (2). More than sixty proteins belong to the MAGE family, but only type I proteins whose expression is restricted to the X chromosome belong to the class of cancer-testicular antigens (3). The first type includes the MAGE-A, -B, and -C subfamilies. MAGE family proteins have a MAGE homology domain (MHD) consisting of approximately one hundred and seventy amino acid residues (4).

The MHD is 46% conserved in the human population. Expression of MAGE-A subfamily proteins in patients with cancer correlates with poor clinical prognosis as well as increased recurrence after therapy (5). MAGE-A subfamily proteins are widely used as target epitopes for immunotherapy because they are present in a large number of tumor types (6). In 2009, the National Cancer Institute (NCI) ranked seventy-five tumor-associated antigens according to characteristics important for selecting an antigen as a target for immunotherapy (e.g. immunogenicity and oncogenicity). MAGE-A3 was ranked eighth (7).

One of the possible approaches to the treatment of MAGE-A3-positive tumors is TCR T-cells. Several clinical trials on the use of high-affinity TCR-T-cells with genetically modified antigen-recognition receptors specific to MAGE-A3 epitopes were prematurely terminated due to lethal outcomes, as the increase in T-cell receptor affinity may lead to off-target activity (8, 9). In the first case, some patients developed severe neurological toxicity due to possible cross-reactivity of the T-cell receptor with a highly homologous epitope of the MAGE-A12 protein (KMAELVHFL), which can be normally expressed in brain cells (8). In the second case, severe cardiac toxicity was observed due to additional recognition of the Titin protein epitope (ESDPIVAQY), which is specific for transverse striated muscles and is found in the myocardium (10).

T-cells during their natural encounters with antigen-presenting Dendritic cells (DCs) can undergo clonal expansion if they recognize the presented peptide (11). Clonal expansion is the process of selection of the most affine T-cell receptor (TCR), i.e. the best TCR clonotype (12). T-cell receptor is composed of two chains: alpha and beta, each of which is, in turn, composed of 4 framework (FR) and 3 complementarity-determining regions (CDR), among which beta-chain CDR3 is the most important as it is mostly responsible for the recognition of the antigen the TCR is specific to.

Previously TCR genomics were facilitated by bulk RNA-seq that had intrinsic inability to properly pair TCR alpha- and beta-chains (13). This insufficiency was later solved by the single-cell multi-omics that currently allow for either targeted or full transcriptome and surface proteome analysis (14), as well as full-length TCR sequencing, proper TCR chain pairing, along with the profiling of transcriptome and proteome of the studied T-cells (15–17).

In this work, we implemented a novel T-cell enrichment protocol (based on the peptide-loaded dendritic cell T-cell induction) to enrich for the MAGE-A3 antigen-specific T-cells (using KVAELVHFL peptide), performed scRNA-seq of the enriched MAGE-A3-specific T-cells, found the Dominant MAGE-A3-specific clonotype, and assessed its effectiveness in an *in vitro* LDH-based cytotoxicity test.

## 2 Materials and methods

### 2.1 Study population

The study population consisted of conditionally healthy adult donors (n = 7) who were preselected for the presence of the HLA-A02 allele via flow cytometry using PE antibodies against human HLA-A02 (343306, Biolegend, United States) and the Attune Nxt flow cytometer (A24858, Thermo Fisher Scientific, Waltham, Massachusetts, United States). The average age of the donors was 27.33 ± 6.34 years (mean ± SE) (male donors *n* = 3, female donors *n* = 4). All donors signed written informed consent to participate in the study.

### 2.2 PBMC isolation

We collected peripheral blood (*n* = 7) in vacuum tubes with EDTA and isolated PBMCs using the Ficoll™ (PanEco, Russia) density gradient centrifugation method. We performed the centrifugation at 400 g at room temperature for 40 min and then collected the buffy-coat cells.

### 2.3 T-cell clonal expansion protocol

We used peptide-loaded dendritic cell (DCs)-based T-cell clonal expansion protocol to induce the single-cell capture-compatible cell number of antigen-specific cytotoxic T lymphocytes *in vitro*. We utilized DCs as they have a unique ability to capture and present antigens in combination with MHC class I and class II molecules for the activation of naïve T-cells, which, in turn, leads to the clonal expansion and differentiation of the naïve T-cells into the effector T-cells (11, 18). In this paper, we developed a multi-stage, maximally optimized, and efficient protocol, based on a previous well-established in-lab protocol that has proven its effectiveness (19–21).

#### 2.3.1 Obtaining DCs

The PBMCs (*n* = 7) were cultivated in RPMI-1640 medium, which was enriched with 10% fetal calf serum (FCS) (Biowest, Nuaillé, France), 2 mM L-glutamine (Biolot, Saint Petersburg, Russia), 5 × 10^-4^ M 2-mercaptoethanol (Sigma-Aldrich, St. Louis, MO, USA), 25 mM HEPES (Biolot, Saint Petersburg, Russia), 80 μg/mL gentamicin (KRKA, Novomesto, Slovenia), and 100 μg/mL benzylpenicillin (Biolot, Saint Petersburg, Russia). This combination is referred to as the culture medium. The obtained PBMCs (80-100 million) were incubated in a culture vial with a surface area of 150 cm² (TPP, Switzerland) at a concentration of 1-2 million cells/mL for 30 minutes in a CO_2_ incubator. The non-adherent cells were then used to isolate CD8+ cells, while the adherent cells were detached from the plastic surface using a cell scraper (TPP, Switzerland). The adherent fraction of PBMCs (20-25 million cells) was cultured in a 150 cm² culture vial (TPP, Switzerland) in the presence of recombinant human granulocyte-macrophage colony-stimulating factor (100 ng/mL, BioLegend, United States) and interleukin-4 (50 ng/mL, BioLegend, United States) for 4 days to generate immature dendritic cells (DCs). Partial media replacement was performed on the 3rd day of culture. On the 5th day, the cells were harvested using a cell scraper, counted, and transferred to 12-well plates (2 mL/well, 1 mL/well). The MAGE-A3 HLA-A02-binding peptide KVAELVHFL peptide (p112-120, Immunotex, Stavropol, Russia) was added to the cell culture at a concentration of 30 μg/mL, followed by the induction of DC maturation on the 6th day using TNF-alpha (25 ng/mL, BioLegend, United States). The HLA-A02 was chosen since it is the most frequent class I HLA genotype among almost all human populations (22).

#### 2.3.2 Obtaining CD8+ T-cells

We used the non-adhesive fraction of cells (*n* = 7) from the previous paragraph for the isolation of CD8+ T-cells via MojoSort™ Human CD8 T-Cell Isolation Kit (480012, BioLegend, United States) according to the manufacturer’s instructions. Immediately after the isolation of CD8+ T-cells, we added IL-7 (581906, BioLegend, United States), IL-15 (570306, BioLegend, United States), and IL-2 (589106, BioLegend, United States) each at a concentration of 10 ng/ml and cultivated the CD8+ cells, at a concentration of 2 million cells/mL, for 6 days. We performed total media replacement on the 3rd day of cultivation and the addition of another dose of IL-2/7/15, alongside anti-CD3 antibody (0.5 μg/ml) (830301, BioLegend, United States) and anti-CD28 antibody (1 μg/ml) (302902, BioLegend, United States). We saved the 6-day-cultivation conditioned media for later use.

#### 2.3.3 DCs and CD8+ T-cells co-culture

Antigen-loaded mature DCs and T-cells were collected from the culture vessel surfaces using a cell scraper and subsequently co-cultured in a new 75 cm² vial (TPP, Switzerland) at a ratio of 1:10 (DCs: CD8+). The first 3 days of co-cultivation were carried out without additional stimulation to selectively eliminate cells that are not receiving stimulation through their T-cell receptor from the mature DCs. On the 4th day of the protocol we added anti-CD3 antibody (0.5 μg/ml) (830301, BioLegend, United States), anti-CD28 antibody (1 μg/ml) (302902, BioLegend, United States), IL-7 (581906, BioLegend, United States), IL-15 (570306, BioLegend, United States), and IL-2 (589106, BioLegend, United States) each at a concentration of 10 ng/ml the co-culture media to maintain cellular viability and promote proliferation of the obtained CD8+ T-cells.

#### 2.3.4 Antigen-specific CD8+ T-cell isolation

On the 8th day of DCs and CD8+ T-cells co-culturing (*n* = 7), we isolated antigen-specific T-cells from the cell co-culture using a two-step isolation process. First, the DCs were eliminated using the MojoSort negative magnetic selection Human CD8 T-cell Isolation Kit (480012, BioLegend, United States). Second, the antigen-specific T-cells were sorted using Flex-T tetramers: we loaded MHC tetramers (HLA-A*02:01) with the MAGE-A3 HLA-A2-binding peptide KVAELVHFL (p112-120) (Immunotex, Stavropol, Russia) and labeled the Flex-T MHC tetramers (BioLegend, United States) with either phycoerythrin (PE) or allophycocyanin (APC) according to the manufacturer’s instructions, and sorted the double-positive (APC-tetramer and PE-tetramer) lymphocytes on a BD FACS Aria I sorter (pressure: 20 psi, mode: “Purity”, speed: 2500 events/sec) (BD Biosciences, Franklin Lakes, New Jersey, United States). The purity of the sorted cell populations ranged from 67% to 88%, yielding between 25,000 to 170,000 antigen-specific cells in total. Additional details can be found in the **Supplementary Materials and Methods** and in **Supplementary Figure 1**.

#### 2.3.5 Stimulation of cell proliferation

Following the CD8 T-cell sorting, we transferred the antigen-specific T-cells (*n* = 7) to a flat-bottomed culture plate at a concentration of 2-4 million cells/mL, where we cultured them for 14 days in the presence of the following T-cell stimulating agents: anti-CD3 antibody (0.5 μg/ml) (830301, BioLegend, United States), anti-CD28 antibody (1 μg/ml) (302902, BioLegend, United States), IL-7 (581906, BioLegend, United States), IL-15 (570306, BioLegend, United States), and IL-2 (589106, BioLegend, United States) each at a concentration of 10 ng/ml. The culture media consisted of equal parts of RPMI-1640 culture media (Biolot, Russia) and the pre-saved CD8+ T-cell conditioned media. The total duration of cultivation of antigen-specific T-cells was 21 days. We visualized the enrichment results in GraphPad Prism 10.2.3 using bar plots.

### 2.4 Sample tag sample barcoding and cell counting for BD Rhapsody single-cell analysis

We incubated cells from different individuals (*n* = 3, other 4 donors were discarded due to the low number of antigen-specific T-cells enriched) with Sample Tag antibodies (BD Biosciences, Franklin Lakes, New Jersey, United States) for 20 minutes at room temperature according to the BD Rhapsody Single-Cell Analysis System User Guide Revision 5.0 (BD Biosciences, Franklin Lakes, New Jersey, United States). After three washing cycles, we stained the cells with Calcein, counted them using the Attune NxT flow cytometer (Thermo Fisher, United States), pooled the samples together, and resuspended them in a cold sample buffer to a final concentration of 60 cells/µl and a volume of 620 µl for loading onto a BD Rhapsody Cartridge. The quality of cell loading into the cartridge was assessed using the InCell Analyzer 6000 with the help of Calcein AM (GE Healthcare, United States).

### 2.5 cDNA library preparation and sequencing

We utilized the BD Rhapsody Express Single-Cell Analysis System (BD Biosciences, Franklin Lakes, New Jersey, United States) for single-cell capture and cDNA library preparation, following the manufacturer’s “TCR/BCR Full Length, Targeted mRNA, and Sample Tag Library Preparation” Protocol. In summary, single cells were captured in the BD Rhapsody cartridge, and magnetic beads were introduced for poly-A mRNA capture. Cells were lysed, and reverse transcription was carried out on the magnetic beads with the captured poly-A mRNA. A template switch oligo was then added, followed by another round of reverse transcription. The Sample Tag cDNA was then denatured, Sample Tag PCR 1 was performed, and bead cDNA was extended using Klenow DNA polymerase fragment. Beads were treated with Exonuclease I, and the cDNA was amplified using TCR primers. The TCR amplicons were denatured and collected, followed by the Human Immune Response Primer Panel on the cDNA (targeting 397 genes with 399 primer pairs) to collect the mRNA panel amplicons. PCR1 products were purified using AMPure XP magnetic beads (A63880, Beckman Coulter, Brea, California, United States) and separated by amplicon size into TCR, mRNA panel, and Sample Tag products. Further amplification and size selection clean-up was carried out on the mRNA and Sample Tag PCR1 products yielding PCR2 mRNA and Sample Tag products. TCR amplicons were normalized to 1.5 ng/μL, followed by random primer extension (TCR RPE) with Klenow DNA polymerase fragment and TCR RPE library clean-up by double-sided selection. Concentrations of PCR2 of mRNA and Sample Tag products and TCR RPE products were measured using Qubit High-Sensitivity dsDNA Kit (Q33231, Thermo Fisher, Waltham, Massachusetts, United States). The final products were then normalized to 4.5 ng/μL for the mRNA panel library and 1.0 ng/μL for the Sample Tag library and the RPE TCR products were used undiluted for the TCR library, and final amplification was performed with Illumina indexes to prepare the libraries. The final libraries were quantified with Qubit 4 and Agilent BioAnalyzer 2100 (Agilent, Santa Clara, California, United States), then pooled (~83/11/5% TCR/mRNA/Sample Tag ratio, estimating 15000 (TCR), 2000 (mRNA), and 1000 (Sample Tag) reads per cell) to a final concentration of 2 nM. Sequencing was performed on a NovaSeq 6000 sequencer (Illumina, San Diego, California, United States) using an SP flow cell with (R1 = 85, R2 = 225, 600 million clusters).

### 2.6 Sequencing data processing

We processed the FASTQ files using the BD Rhapsody pipeline v1.12 (BD Biosciences, Franklin Lakes, New Jersey, United States). The pipeline first filtered out low-quality read pairs based on criteria such as read length, highest single-nucleotide frequency, and mean base quality score. It then analyzed the remaining high-quality R1 reads to identify cell label and unique molecular identifier (UMI) sequences. High-quality R2 reads were aligned to the reference panel sequences (mRNA) using Bowtie2. Reads with the same cell label, UMI sequence, and gene were collapsed into single molecules. UMI counts were adjusted using error correction algorithms — recursive substitution error correction (RSEC) and distribution-based error correction (DBEC) — to mitigate errors from both sequencing and PCR. Cell counts were estimated using second derivative analysis to filter out noise cell labels; only cell labels beyond a single observed inflection point were considered valid. The pipeline then used the sample tags (single-cell multiplexing kit; BD Biosciences) for sample demultiplexing and to exclude multiplets, identifying a total of 5.491 single cells. Following this, the pipeline aligned TCR RPE library reads on a per-cell basis to create TCR contigs, annotated these contigs, and generated gene expression (gene/cell) matrices (GEX matrices) for each biological sample. Additionally, a cumulative Adaptive Immune Receptor Repertoire (AIRR) matrix was created for the TCR contigs.

### 2.7 ERGO-II TCR-peptide-MHC affinity prediction

We downloaded the ERGO-II neural network repository (23) and initiated the tool from the terminal with the selection of the input file and database (McPAS-TCR) (24). McPAS-TCR is a manually curated database based on published literature containing information on over twenty thousand T-cell receptor sequences, the antigens they bind to, T-cell type (CD4+/CD8+), and MHC type (MHC-I/MHC-II). McPAS-TCR includes information about T lymphocytes that expand in various human or mouse pathological conditions (including viral infections, cancer, and autoimmune reactions). The CSV input file for ERGO-II (a pre-ERGO-II-generated input file, (25, pre ERGO-II.ipynb) contains information about TCR CDR3α and CDR3β sequence, peptide sequence, MHC type (MHC-I/MHC-II class), V and J genes, and T-cell type (CD4+/CD8+), the pre-ERGO-II-generated input file also contained single cell indices for later data frame merging. The output file contains a prediction value for the score of T-cell receptor binding to the peptide/MHC complex, which ranges from 0 to 1, where 0 is the minimum or no affinity and 1 is the maximum affinity.

### 2.8 TCRscape clonotype selection

We imported the GEX matrices of each biological sample, the multi-sample AIRR matrix, and the ERGO-II output file into TCRscape (25), merged the GEX matrices, performed “Counts Per Million” data normalization, replaced the zeroes in the data frame with the ones, log2-transformed the data, gated CD8+ T-cells, counted dominant full-length T-cell receptor clonotypes of the gated T-cells, created a merged data frame containing the gene expression of the key T-cell markers (*CD4, CD8, FOXP3*), clonotype information and ERGO-II-generated binding scores, performed Principal Component Analysis (PCA) on the merged data frame, assessed the dimensionality of the merged data frame by Scree plot, and performed Uniform Manifold Approximation and Projection (UMAP) dimensionality reduction using the first 4 principal components.

### 2.9 Plasmid construction and lentivirus preparation

We generated TCR-containing transfer plasmid, VSV-G encoding plasmid, and two third generation lentivirus packaging plasmids (containing Gag-Pol and Rev genes respectively) in E. coli NEB Stable strain (C3040I, New England Biolabs, Ipswich, Massachusetts, United States), and verified the resulting plasmids using restriction enzymes and gel electrophoresis. We then delivered the above plasmids using Lipofectamine 2000 (11668500, Thermo Fisher, Waltham, Massachusetts, United States) into HEK-293T packaging cells generously provided by Dr. Hiroshi Shiku (Mie University, Japan).

### 2.10 Lentivirus concentration and titration using qPCR

We concentrated the produced lentiviruses using the commercial TransLv™ Lentivirus Precipitation Solution (5×) (FV101-01, TransGen, China) and titrated them using quantitative (with dilution standards) PCR for proviral DNA (TransLv™ Lentivirus qPCR Titration Kit) (FV201-01, TransGen, China) in HEK-293T.

### 2.11 TCR T-cell manufacturing

To obtain TCR T-cells specific for MAGE-A3, Retronectin (Sci Store, Russia) at a concentration of 25 µg/mL and anti-CD3 antibodies (BioLegend) at 5 µg/mL in ACDA (citrate buffer with glucose) were adsorbed one day prior to the experiment. This solution was applied to the wells of a 12-well plate at 415 µL per well. CD3+ T-cells were isolated from the PBMCs (*n* = 7) of HLA A02-positive healthy donors using MojoSortTM Human CD3 negative magnetic Selection Kit (480134, Biolegend, United States). The isolated cells (0.75 million/mL) were then incubated in the plate with retronectin and anti-CD3 antibodies, supplemented with IL-2 (300 units/mL, Biotech LLC, Russia), for 48 hours.

The day before transduction, Retronectin (Sci Store, Russia) was adsorbed at a concentration of 25 µg/mL in ACDA. This solution was applied to the wells of a 24-well plate at 255 µL per well. On the day of transduction, cells were harvested, centrifuged, resuspended in a serum-free medium, and counted. Then, 200,000 cells were transferred into Retronectin-coated wells at a volume of 500 µL/well. After that, to each well we added lentivirus (200,000 particles/well, i.e. at a multiplicity of infection (MOI) of 1) and protamine sulfate (5-8 µg/mL) to enhance transduction (26). The plate was then centrifuged for 2 hours at 600 x g and 32°C. After centrifugation, 500 µL of warm serum-free medium containing IL-2 (final concentration 300 units/mL) was added to the cells that were allowed to incubate overnight. The following morning, cells were transferred into the wells of a 12-well plate with an equal volume of complete medium containing IL-2. Cell growth, conglomerate formation, and nutrient medium condition were monitored visually, and growth factors were refreshed every two days.

### 2.12 *In vitro* TCR T-cell assessment in a cytotoxicity test

To assess cytotoxic activity against tumor cells, transduced or vehicle-transduced (with a lentivirus without transfer plasmid) cells were harvested, centrifuged at 350 g for 10 minutes, and counted. Tumor cells, in the logarithmic phase of growth, were harvested using a 1:3 mixture of trypsin (0.25%) (PanEco, Russia) and Versen solution (Vector, Russia). Tumor cells were seeded into a 96-well flat-bottom plate at a concentration of 5,000 cells/well. T-cells (50,000 cells/well) were added 2-3 hours later, resulting in an effector-to-target ratio of 10:1. The co-culture was allowed to incubate for 16-18 hours in a medium containing 5% FCS. Forty-five minutes before the end of the incubation period, 10 µL of 10X lysing solution was added per 100 µL of cell suspension to control the maximum release of lactate dehydrogenase (LDH) from the cells.

After completing the lysis, the cell plate was centrifuged at 250 × g for 4 minutes to gently pellet the cells. Aliquots of 50 µL from each well were then transferred to a new 96-well flat-bottom plate for the immunoassay. To each well of cell culture supernatants, 50 µL of reconstituted lactate dehydrogenase (LDH) enzyme-substrate mixture was added. The plate was covered with foil or an opaque cover slip to protect it from light and was incubated for 30 minutes at room temperature. After the incubation, the reaction was stopped with 1M acetic acid solution. Optical density was measured at 490 or 492 nm immediately after stopping the reaction. Cytotoxic activity was calculated in doubles using the following formula:


$$\%\;Cytotoxicity=\frac{OD\;\left(T-cells\;+\;targets\right)-OD\;\left(T-cells\right)}{OD\;\left(maximal\;tumor\;lysis\right)-OD\;\left(spontaneous\;tumor\;lysis\right)}\times 100$$

We then analyzed the per-sample-averaged cytotoxicity data in GraphPad Prism 10.2.3 using one-way ANOVA with Tukey correction for multiple testing.

## 3 Results

### 3.1 T-cell clonality expansion

**Figure 1:**
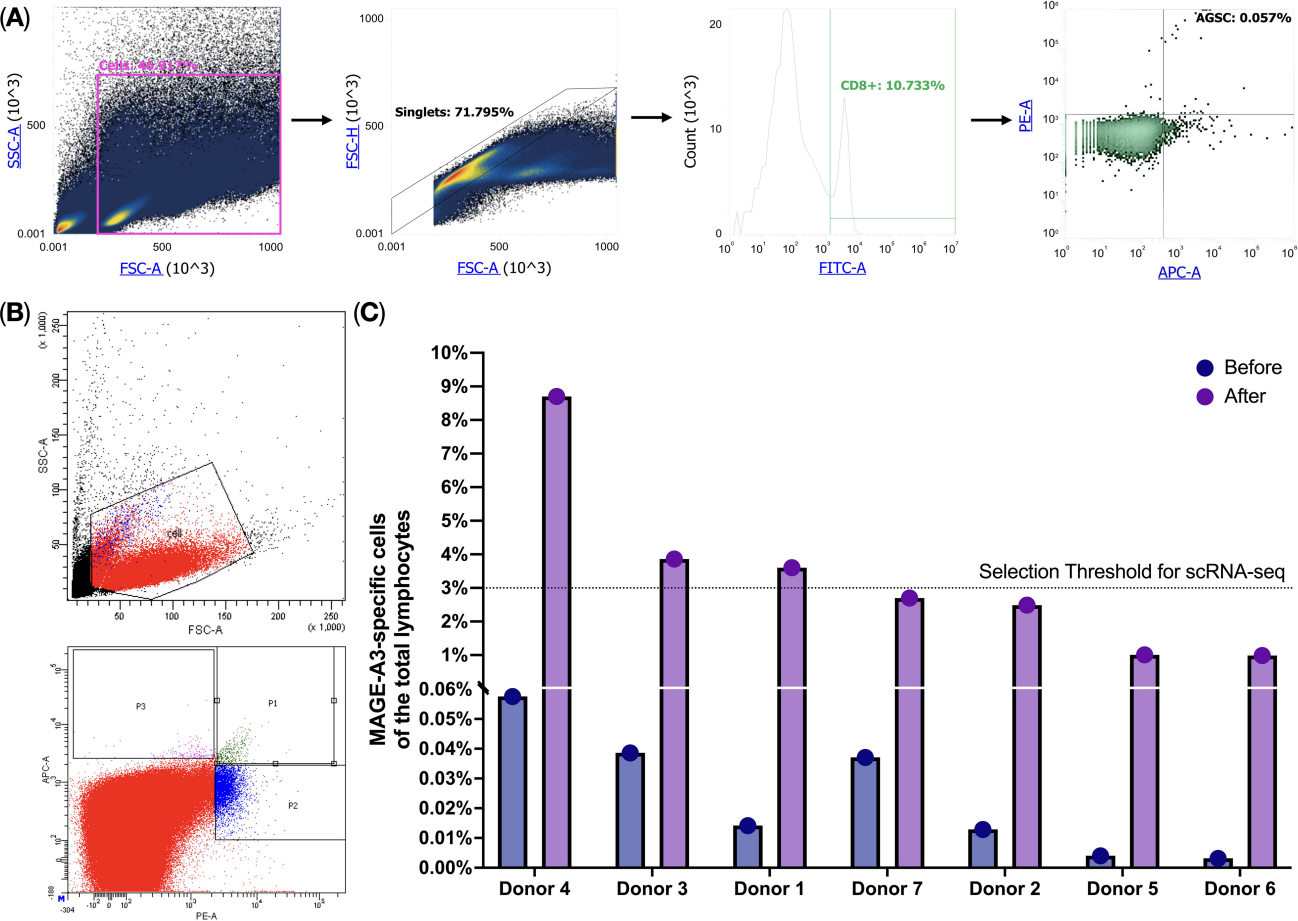
MAGE-A3-specific T-cell flow cytometry analysis. **(A)** HLA-A02-positive donor screening for MAGE-A3-specific T-cells; **(B)** MAGE-A3-specific T-cell enrichment after the cultivation protocol; **(C)** MAGE-A3-specific T-cell before and after the cultivation protocol.

We performed a Dendritic cell-based antigen-specific T-cell induction protocol on PBMCs (*n* = 7) and observed an increase (mean fold change = 191.0, SD = 87.9) in the presence of MAGE-A3-specific T-cells after the protocol. The percentage of the MAGE-A3-specific T-cells isolated from healthy donors (*n* = 7) was 0.02% ± 0.015 (average ± SD) of the total lymphocytes, after applying the protocol, this percentage increased to 3.33% ± 2.61 (average ± SD) (**Figure 1**). We have selected donors with the enrichment of the MAGE-A3-specific T-cells above 3% for single-cell multi-omics analysis that we performed on a BD Rhapsody platform using the “TCR/BCR Full Length, Targeted mRNA, and Sample Tag Library Preparation” Protocol.

### 3.2 TCR clonotype selection

**Figure 2:**
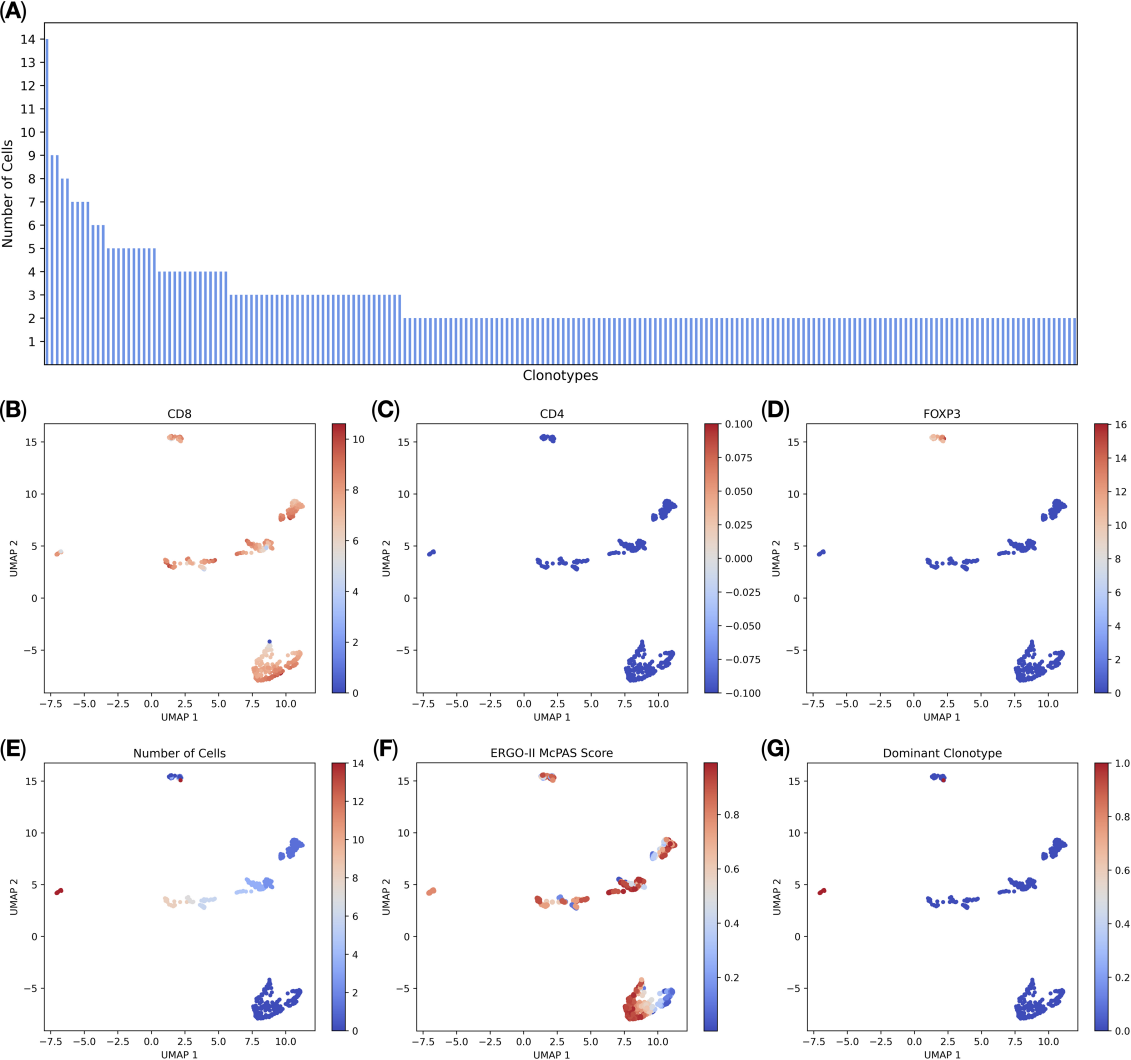
T-cell multi-omic analysis (*n* = 3, 5.491 single cells). **(A)** Distribution of complete T-cell receptor clonotype sequences. Each bar on the x-axis represents a clonotype, the y-axis shows the number of cells per clonotype, all clonotypes with 2 or more cells per clonotype are shown; **(B)** normalized *CD8* gene expression; **(C)** normalized *CD4* gene expression; **(D)** normalized *FOXP3* gene expression; **(E)** the number of cells per clonotype; **(F)** ERGO-II-predicted binding scores to the target peptide; **(G)** the Dominant clonotype (with 14 cells per clonotype) is shown in red.

We imported the BD Rhapsody-generated T-cell multi-omic data into TCRscape and identified 3000 T-cell receptor clonotypes, among which 191 clonotypes were present in 2 or more cells (**Figure 2A**). We were also able to perform post-sequencing quality control of the CD8+ T-cell sorting (99,9% of the T-cells were CD8+ T-cells) (**Figure 2B**). We used two main criteria for the Dominant clonotype selection: cell count per clonotype (main criterion) and predicted binding score towards the target peptide (secondary criterion). We identified a single dominant clonotype that was expressed by 14 cells (**Figures 2A, E, F**) and was predominantly represented by *CD8+ CD4−FOXP3−* T-cells (**Figures 2B–D, G**). We also observed that the dominant clonotype had a medium predicted binding score (i.e. affinity) towards the KVAELVHFL peptide, which is characteristic of the naturally occurring cytotoxic T-cells (27–29).

### 3.3 Plasmid construction

We then designed the insert for the lentiviral transfer plasmid. The insert included the TCRa and TCRb sequences in a single reading frame, separated by a signal to reset the polypeptide with the P2A polypeptide (See **Supplementary Figure 2**), the insert was then cloned into the pLenti hPGK GFP vector replacing the GFP gene (See **Supplementary Figure 3**).

### 3.4 Cytotoxicity assay

**Figure 3:**
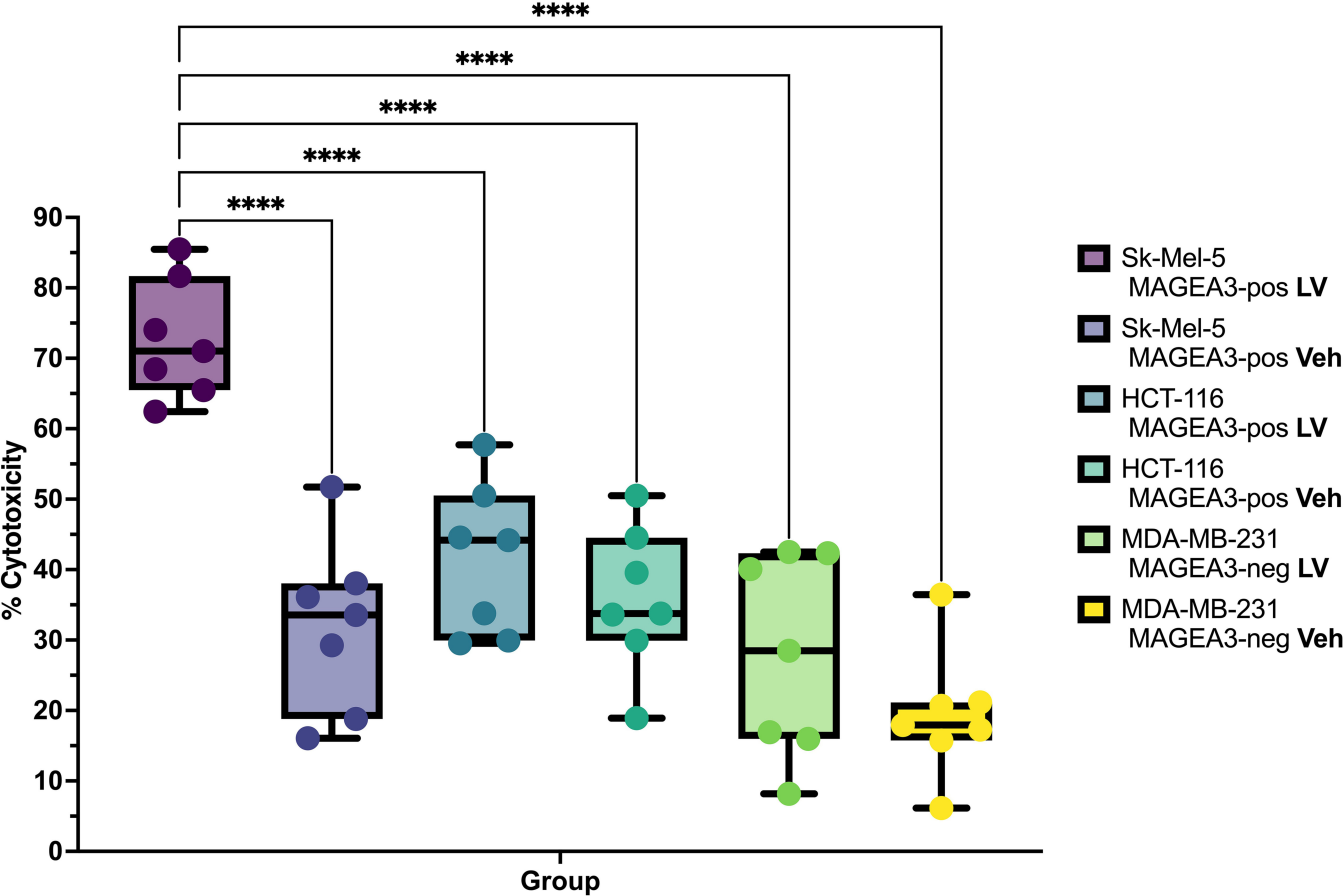
Cytotoxicity percentage and specificity of the candidate TCR against different tumor cell lines (*n* = 7), LV – lentiviral transduction (TCR-encoding), Veh– lentiviral transduction (Vehicle). **** - q-values < 0.00005.

To evaluate the efficiency of the candidate TCR in targeting and eliminating MAGE-A3+ tumor cells, we engineered TCR T-cells via transduction of the anti-CD3-treated PBMCs with a lentivirus containing the aforementioned anti-MAGE-A3 construct (n=7), with the vehicle-transduced cells serving as a control. We then co-cultured the TCR T-cells and the non-transduced cells with the MAGE-A3-high SK-MEL-5, the MAGE-A3-low HCT-116, and the MAGE-A3-negative MDA-MB-231 cell lines. The LDH cytotoxicity assay results revealed high cytotoxicity against the SK-MEL-5 cell line (**Figure 3**, **Table 1**), indicating that the candidate TCR is effectively recognizing and targeting cells that express the MAGE-A3 antigen. Similar trends were observed in response to HCT-116 cells, which have lower levels of MAGE-A3 antigen expression, which aligns with the findings reported by Xiang Zhao et al. (30).

**Table 1:** Cytotoxicity percentage and specificity of the candidate TCR against different tumor cell lines (*n* = 7), LV, lentiviral transduction (TCR-encoding); Veh, lentiviral transduction (Vehicle).

	Donor 1	Donor 2	Donor 3	Donor 4	Donor 5	Donor 6	Donor 7	Mean	SD
**Sk-Mel-5** **MAGEA3-pos LV**	68,46	71,02	65,48	62,43	74,02	85,48	81,67	72,65	8,41
**Sk-Mel-5** **MAGEA3-pos Veh**	51,72	38,04	18,80	36,11	16,05	29,26	33,56	31,94	12,12
**HCT-116** **MAGEA3-pos LV**	33,75	44,16	44,52	29,52	29,92	57,69	50,50	41,44	10,78
**HCT-116** **MAGEA3-pos Veh**	33,75	44,52	29,92	50,50	18,93	33,62	39,55	35,83	10,28
**MDA-MB-231** **MAGEA3-neg LV**	42,33	42,50	40,06	15,97	8,18	16,93	28,48	27,78	14,27
**MDA-MB-231** **MAGEA3-neg Veh**	36,45	20,65	6,16	15,75	21,13	17,29	17,96	19,34	9,04

## 4 Discussion

In this paper, we performed a complex pipeline for the induction of MAGE-A3 antigen-specific T-cells: starting with the T-cell enrichment using peptide-loaded DCs, followed by single-cell RNA sequencing of the enriched T-cells on the BD Rhapsody platform, T-cell clonotype analysis was then performed via TCRscape, a clonotype discovery tool tailored to the BD Rhapsody data, which resulted in the identification of the dominant clonotype followed by the assessment of its effectiveness *in vitro* via an LDH cytotoxicity test.

We observed a successful T-cell clonality expansion after our protocol (mean fold change = 191.0, SD = 87.9), with sufficient T-cell numbers that allowed us to successfully perform scRNA-seq of such cells. This validates the described protocol as a suitable approach for the discovery of antigen-specific T-cells and their TCRs using modern single-cell analysis methods.

By utilizing single-cell sequencing technology, we obtained detailed information about the sequence of each TCR, enabling us to accurately construct the obtained TCR clones, as well as, information about the immune transcriptome of each T-cell, allowing us to assess the functionality state of the cells and enhance the selection process of candidate TCR clones. This provides a significant advantage over currently used methods like bulk RNA-seq, which do not provide any cell-of-origin information for sequenced TCRs making it impossible to determine which alpha and beta chains originated from the same T-cell, or multiplex PCR and 5’-RACE approaches, which exhibit lower accuracy and sensitivity (31).

Our analysis of the BD Rhapsody-generated single-cell multi-omic data in TCRscape revealed 191 unique clonotypes that were detected in 2 or more T-cells, thus also confirming the successful enrichment of the T-cells using our T-cell enrichment protocol.

Cytotoxicity results also validated our approach for clonotype selection, as TCR T-cells transduced with the dominant clonotype-lentivirus show potent cytotoxicity. This shows that the observed number of cells per clonotype could be a valid criterion for the *in-silico* search for the effective clonotype.

However, the small sample size constitutes a limitation of this study, as the data might not fully represent the overall population. Nevertheless, the findings remain highly relevant for TCR T-cell therapy, given that we identified a strong dominant clonotype with a 14-fold expansion compared with the baseline TCR occurrence, highlighting the potential efficacy of our approach even within a small cohort. Such results underscore the advantage of single-cell multi-omics technologies, which enable significant data acquisition and meaningful results even with limited sample sizes.

Previous efforts for the enrichment and isolation of antigen-specific T-cells have been extensively explored across various fields, including infectious diseases, autoimmunity, and cancer. For example, Klinger et al. (32) pioneered a multiplex approach combining immune assays with receptor sequencing to identify antigen-specific TCRs, laying the groundwork for subsequent research. Similarly, Sharma et al. (33, 34) demonstrated the utility of TCR repertoire analysis monitoring transplant patients and autoimmune diseases, underscoring the broader applicability of such pipelines beyond oncology. Furthermore, Dziubianau et al. (35) provided further evidence of the importance of precise TCR selection processes through their work on enriching antigen-specific T-cells in viral infections. Collectively, these methodologies underscore the critical need to identify effective TCR candidates, a goal that aligns directly with the objectives of our current study, especially when integrated with single-cell sequencing.

In conclusion, we have modified a dendritic cell-bases protocol to be efficient for T-cell clonal expansion, obtained a potent TCR via single-cell sequencing, and successfully tested it in an *in vitro* cytotoxicity test against a MAGE-A3-positive tumor. Nevertheless, further investigation is required to determine the applicability of our results *in vivo*.

## Data Availability

The original contributions presented in the study are publicly available. This data can be found via the Zenodo repository: accession number 12804940.
