# Supplementary material for: Modified Dendritic cell-based T-cell expansion protocol and single-cell multi-omics allow for the selection of the most expanded and in vitro-effective clonotype via profiling of thousands of MAGE-A3-specific T-cells
Source: Front Immunol. 2024 Oct 10;15:1470130. doi: 10.3389/fimmu.2024.1470130 (PMC11499154; doi:10.3389/fimmu.2024.1470130)
Supplement: Supplementary file 1 [file DataSheet1.docx]

Supplementary Material

# Supplementary Materials and Methods

## Antigen-specific CD8+ T-cell Isolation

Staining was conducted according to the manufacturer's protocol. Cells exhibiting simultaneous fluorescence in both PE and APC channels (double positive) were identified as antigen-specific cells. Unstained cells and cells stained with Flex-T tetramers not conjugated to any peptide served as controls (Supplementary Figure 1).

-

**Supplementary** **Figure 1**. Flex-T tetramer staining and sorting results on a BD FACS Aria I flow cytometer and cell sorter. (**A**) Cells stained with Flex-T tetramers not conjugated to any peptide.
(**B**) Cells stained with Flex-T tetramers conjugated with KVA peptide.
(**C**) Post-sorting results of cells stained with Flex-T tetramers conjugated with KVA peptide.

# Supplementary Figures and Tables

## Supplementary Figures


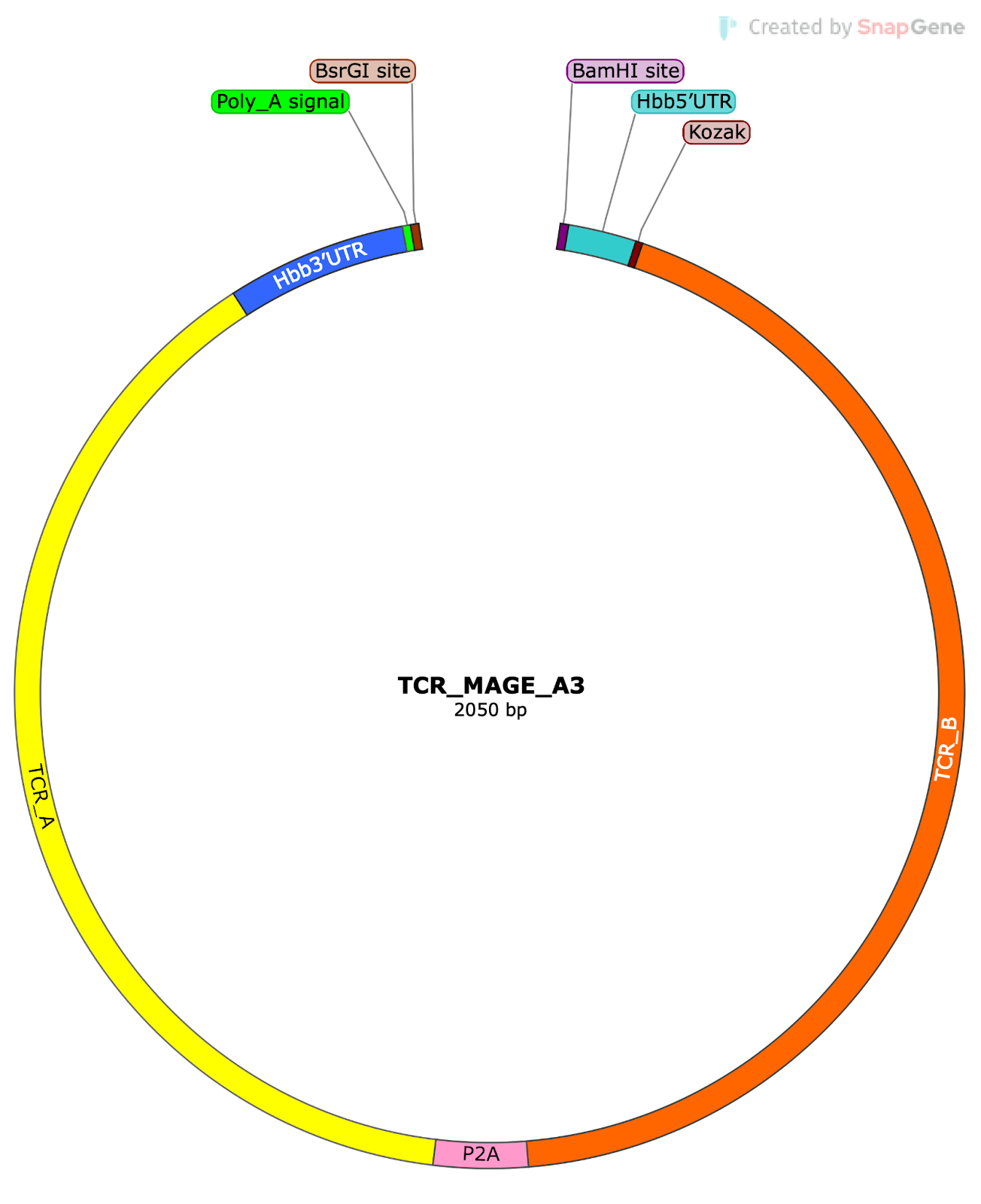


**Supplementary** **Figure 2.** The lentiviral transfer plasmid insert encoding the dominant MAGE-A3-specific TCR.


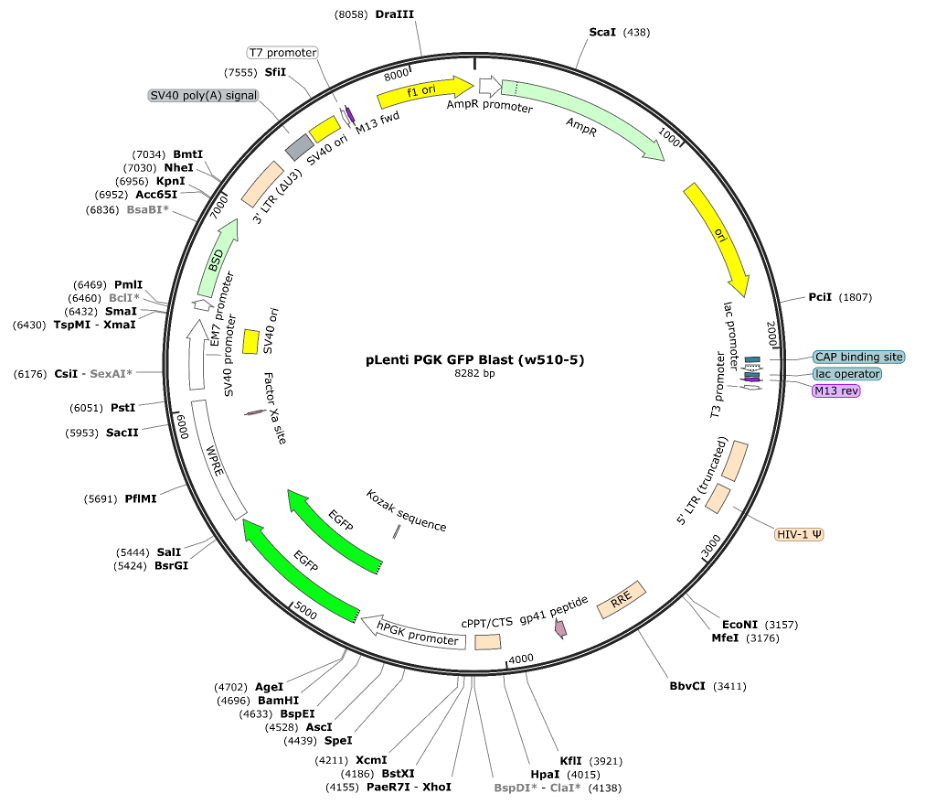


**Supplementary** **Figure 3.** The pLenti hPGK GFP lentiviral transfer plasmid
